# Do Social Networks Differ? Comparison of the Social Networks of People with Intellectual Disabilities, People with Autism Spectrum Disorders and Other People Living in the Community

**DOI:** 10.1007/s10803-014-2279-3

**Published:** 2014-10-18

**Authors:** A. E. van Asselt-Goverts, P. J. C. M. Embregts, A. H. C. Hendriks, K. M. Wegman, J. P. Teunisse

**Affiliations:** 1 Faculty of Health and Social Studies, HAN University of Applied Sciences, P.O. Box 6960, 6503 GL Nijmegen, The Netherlands; 2 Tranzo, Tilburg University, Tilburg, The Netherlands; 3 Dichterbij Innovation and Science, Gennep, The Netherlands; 4 Medical and Clinical Psychology, Tilburg University, Tilburg, The Netherlands; 5 School of Educational Science, Faculty of Social Sciences, Radboud University, Nijmegen, The Netherlands; 6 MEE Oost-Gelderland, Doetinchem, The Netherlands; 7 Dr. Leo Kannerhuis, Doorwerth, The Netherlands

**Keywords:** Intellectual disabilities, Autism, Social network, Satisfaction, Wishes

## Abstract

The aim of this study was to determine the similarities and differences in social network characteristics, satisfaction and wishes with respect to the social network between people with mild or borderline intellectual disabilities (ID), people with autism spectrum disorders (ASD) and a reference group. Data were gathered from 105 young adults living independently in the community. The social networks of people with ID and ASD are more restricted than those of the reference group. Compared with the other groups, people with ASD are less often satisfied with their networks. Each group has its own characteristics, issues and wishes with respect to their social network. Practical measures to enable professionals to adapt to these issues are discussed.

## Introduction

According to the United Nations Convention of the Rights of Persons with Disabilities, people with disabilities have the right to live in the community with choices equal to others (United Nations 2006; Hewitt et al. 2013). This right is translated into policy worldwide, for instance in the United States in creating opportunities for community living (Hewitt et al. 2013) and in the United Kingdom, where people with disabilities are considered as citizens participating in all aspects of community and in control of the decisions in their lives (Department of Health 2009). In the Netherlands—under the influence of the Dutch Social Support Act (Wet maatschappelijke ondersteuning 2007)—more and more vulnerable people (e.g., elderly people or people with disabilities or disorders) are living independently in the community with the aim to participate in society (de Klerk et al. 2010; Lub et al. 2010). Physical presence in the community, however, does not guarantee real social inclusion, just as taking part in an activity does not guarantee meaningful social contact (Ager et al. 2001). Real inclusion means supporting people to become connected, be part of the place or activity and belong (Gomez 2013). Instead of moral imperatives of mainstreaming and independent living for all, meaningful activity and social relationships are needed to become someone instead of be placed somewhere (Clegg et al. 2008). Research shows that professionals play an important role in facilitating social inclusion by mapping these social networks and supporting the person in expanding or strengthening his or her social network, if required (e.g., Abbott and McConkey 2006; van Asselt-Goverts et al. 2014a). To achieve this, it is important to investigate the social networks of these vulnerable people living in the community. What are the characteristics of their social networks? How satisfied are they with their networks and what are their wishes with respect to them? In this article, we focus on two specific groups: high-functioning adults with autism spectrum disorders (ASD) and adults with mild intellectual disabilities (ID), because both these target groups experience difficulties in developing and maintaining social contacts. We compare the networks of these two groups with one another and with the networks of a reference group. Although people with ASD and ID both have limitations with respect to social contact, the nature and consequences of these limitations differ.

In the Diagnostic and Statistical Manual of Mental Disorders—Fifth Edition (DSM-5), autism spectrum disorder is characterized by two core symptoms: (a) deficit in social communication and social interaction and (b) restricted, repetitive behaviours, interests or activities (American Psychiatric Association 2013). Three severity levels are defined, based on the amount of support needed due to these symptoms, which underlines the importance of social networks. Given the deficit in social communication and social interaction, people with ASD face significant difficulties in developing and maintaining contacts with network members (American Psychiatric Association 2013; Friedman et al. 2013; Orsmond et al. 2004). However, research on social networks of adults with ASD is scarce (Orsmond et al. 2004). The existing research focuses mainly on the social networks of children (e.g., Bauminger et al. 2008; Bauminger and Kasari 2000; Kasari et al. 2011) and adolescents (e.g., Lasgaard, et al. 2010; Locke et al. 2010; Whitehouse et al. 2009), or on social support of the parents of children with ASD (e.g., Ekas et al. 2010; Siman-Tov and Kaniel 2011; Weiss et al. 2013). Research shows that high-functioning children with autism report having at least one friend, but also that they are lonelier and have less complete understandings of loneliness compared to typically developing children (Bauminger and Kasari 2000). These children perceive their friendships as less close, helpful and intimate (Bauminger et al. 2008). The majority of these children are at the periphery of their network at school and have poorer quality friendships and fewer reciprocal friendships (Kasari et al. 2011). Similar findings are reported for high-functioning adolescents with ASD: they feel lonelier (Lasgaard et al. 2010; Locke et al. 2010; Whitehouse et al. 2009), report poorer quality of their best-friendship (Whitehouse et al. 2009) and are socially isolated or at the periphery of their network at school (Friedman et al. 2013; Locke et al. 2010). Longitudinal research suggests some improvements of social behaviour when children with ASD reach adolescence and adulthood (Seltzer et al. 2003, 2004). However, cross-sectional research comparing adults with adolescents suggests that adults have more impairments in social interaction and have fewer peer relationships than adolescents (Orsmond et al. 2004; Seltzer et al. 2003). Social deficit is persistent and social isolation remains in adulthood (e.g., Friedman et al. 2013; Seltzer et al. 2004). Approximately one quarter to one-third of adults with ASD report having at least one friendship (Eaves and Ho 2008; Howlin et al. 2004) and the same percentage report spending time with others in consequence of their hobby, or attend a club or church regularly (Eaves and Ho 2008). Although high-functioning adults with ASD do have friendships, their relationships are less close, less empathic, less supportive and less important to the individual, compared to people without ASD (Baron-Cohen and Wheelwright 2003). However, perceived informal social support is related to quality of life (Renty and Roeyers 2006) as well as marital adaptation (Renty and Roeyers 2007) in adults with ASD. To our knowledge, a more comprehensive examination of structural (e.g., size and composition, frequency of contact, initiation of contact, length of the relationship) and functional (e.g., perceived emotional and practical support) characteristics of the social network of adults with ASD from their own perspective is lacking.

In the field of ID more research is conducted regarding social networks than in the field of ASD. With respect to the structural characteristics of social networks of people with ID, research mainly focuses on the number of network members. In their systematic review Verdonschot et al. (2009) concluded that the social networks of people with ID are often small, but the size in the research literature varies from a median of six network members (Robertson et al. 2001) to an average of 11.67 (Lippold and Burns 2009), 14.21 (van Asselt-Goverts et al. 2013) and 22 (Forrester-Jones et al. 2006) for people with ID living in the community. Differences between studies in the size of the social networks of people with ID might be attributable to the use of different measures: the MSNA (Baars 1994; van Asselt-Goverts et al. 2013), the Social Network Map (Robertson et al. 2001; Tracy and Abell 1994), the Social Network Guide (Forrester-Jones et al. 2006), or the Social Support Self Report (Lippold and Burns 2009). Moreover, the observed variation in the size of the social networks reported between studies could be contributed by the design of the study with respect to the informants: the people with ID themselves (van Asselt-Goverts et al. 2013; Forrester-Jones et al. 2006; Lippold and Burns 2009) versus proxy informants, such as support staff (Robertson et al. 2001). With respect to the functional characteristics, research indicates that social support is perceived mainly from professionals (Forrester-Jones et al. 2006) and that professionals are highly appreciated by individuals with mild ID; for affection comparable with family and acquaintances and for practical/informational support, they are valued even higher (Van Asselt-Goverts et al. 2013). Moreover, the majority of the participants (73.1 %) are satisfied with their social networks and improvement in the area of strengthening existing ties (e.g., more frequent contact, better contact) is desired, as opposed to expansion of the network (van Asselt-Goverts et al. 2014b). However, these data on both structural and functional characteristics are difficult to interpret because normative data are lacking (van Asselt-Goverts et al. 2013). Even though several researchers have used different groups, most of the times the groups consisted only of people with ID (e.g., difference in age, degree of ID or living accommodation). In one study, people with ID were compared to people with physical disability (PD; Lippold and Burns 2009), finding that people with ID had more restricted social networks than people with PD, despite being involved in more activities. Widmer et al. (2008) compared individuals with ID, individuals with ID and psychiatric disorders and students matched for age and sex, but only with respect to the family network. Compared with the control group, people with ID less often consider themselves or their family members as sources of emotional support (Widmer et al. 2008).

From this we can conclude that data on the social networks of high-functioning adults with ASD are lacking. Moreover, data on the social networks of people with ID are hard to compare because of differences in methods of data collection (i.e., with respect to measures used and choice of participants) and the lack of normative data. We therefore hypothesized that the networks of people with ASD (Friedman et al. 2013; Seltzer et al. 2004) and the networks of people with mild ID (e.g., Lippold and Burns 2009; Robertson et al. 2001; Verdonschot et al. 2009) are smaller than those of other people living in the community. However, the number of network members is not a decisive factor in well-being (Lippold and Burns 2009). In consequence, as well as the usual quantitative approach, focussing on the size of the network, we also used a more qualitative approach, including crucial structural and functional network characteristics ranging from the frequency of social contacts to practical and emotional support (Baars 1994; van Asselt-Goverts et al. 2013). Moreover, how people themselves perceive their networks is essential (van Asselt-Goverts et al. 2014b). Because people with ASD and ID experience difficulties in developing and maintaining social contacts, we focus in this study on their description and their opinions of their networks. Therefore the objective of this study was to determine the specific network characteristics of people with ID and ASD and their specific opinions regarding their networks. Specific research questions were:

1. Are there differences between people with ASD, mild ID and a reference group in their description of structural network characteristics (i.e., size, frequency, length and initiation)?
2. Are there differences between these three groups in their description of functional network characteristics (i.e., affection, connection, preference and practical/informational support)?
3. Are there differences in how the three groups perceive their social network (i.e., satisfaction and wishes)?

## Methods

### Participants

Participants met the inclusion criteria if they were young adults, living independently in the community for at least 2 years (i.e., lived in the community alone, with a partner, friend or children; persons living in a group home or with their family were thus excluded from the present study). Moreover, included participants were adults with a mild to borderline ID or adults with ASD and without ID or adults with neither of those disabilities/disorders. The persons with ID were recruited via 7 care organizations which were located in the southeast of the Netherlands. The persons with ASD were recruited from two MEE support agencies (organisations that provide mobile advice and support to people with disabilities), located in the east and middle of the Netherlands. The reference group subjects (REF group; i.e., people without ID or ASD) were living in the southeast of the Netherlands and were recruited by students of the HAN University of Applied Sciences. The students were asked to recruit two participants, taking account of age and gender, with respect to the REF group. These two participants were each interviewed by another student who had not been involved in the recruitment. The total sample consisted of 105 persons: 33 persons with mild to borderline ID, 30 persons with ASD and 42 persons in the REF group. The age of the participants varied from 19 to 36 years for both ID and REF group and 19–37 years for the ASD group. The mean age of the participants of the distinct groups did not differ significantly, for the ID group 28.9 (SD = 5.2), for the ASD group 29.7 (SD = 4.7) and for the REF group 28.4 (SD = 4.8), *F*(2, 102) = 0.702, *p* = .498. Although the proportion of men in the ASD group seemed higher, this was not a significant difference (see Table 1). Although the three groups were thus matched for age and gender, Table 1 shows that for having an intimate relationship, living situation and work situation the groups did differ significantly. Further analyses showed that the participants of the REF group had a partner significantly more often and lived with this partner and/or their children than both other groups. They also more often had work or outdoor activities during the day. The differences between ID and ASD were not significant on these demographic characteristics.

**Table 1:** Demographic characteristics (%) of participants in the ID, ASD and REF group compared

	ID (*n* = 33)	ASD (*n* = 30)	REF (*n* = 42)	*χ* ^*2*^	*p*
Gender (% male)	48.5	66.7	45.2	3.514	.173
Intimate relationship (% partner)	51.5	53.3	85.7	12.451	.002
Living situation (% living together)^a^	30.3	46.7	81.0	20.422	.000
Work situation (% work and outdoor activities)^b^	78.8	60.0	95.2	13.626	.001

^a^With partner and/or children; ^b^ a job, supported employment, sheltered workshop, day activity program or school

### Measures

#### Maastricht Social Network Analysis

The structural and functional characteristics of the social networks of the participants in this study were mapped in an interview using the Maastricht Social Network Analysis (MSNA; Baars 1994). With the MSNA important network members were listed on three cards; one for family members (e.g., partner, parents, siblings and other family members), one for acquaintances (e.g., friends, colleagues, neighbours, other acquaintances) and one for professionals (e.g., support staff, therapists, social workers, coaches). Each member of the network of family and acquaintances was then scored on 20 items. For family and acquaintances, items included structural characteristics (e.g., demographic characteristics, frequency of contact, length of the relationship, initiation of contact) and functional characteristics (e.g., the supportiveness of the contact). The functional characteristics were operationalized along four dimensions: affection (e.g., feeling safe and secure with the person, loving the person), connection (e.g., liking the same things), preference (e.g., preference for contact with the person, liking the contact), and practical/informational support (e.g., being helped by the person when you don’t know something or aren’t able to do something). Each dimension was measured by one question per network member. For professionals only ten characteristics were used in the MSNA (e.g., frequency of contact, length of the relationship, initiation of contact and functional characteristics), because the other items were less relevant with respect to them (e.g., demographic characteristics). In this study we present the characteristics which are relevant for all groups of network members (e.g., size and composition of the network, frequency of contact, initiation of contact, length of the relationship and the functional characteristics).

To ensure a minimum of reliability and validity for the MSNA, the following were taken as starting points: (a) only information on network members with whom there was a direct connection should be provided; (b) the information obtained in such a manner was of a largely objective, factual nature; and (c) only information which was known for certain was provided, with anything that was uncertain therefore omitted (Baars 1994).

For the present study, the original form of the MSNA was adapted for use with people with mild ID by simplifying questions and using visualization. This variation was used for all participants, including for participants in the ASD and REF groups. First, a genogram (i.e., family tree) was used to map the characteristics of the participant’s family relations. Second, an ecogram was created to visualize the remainder of the social network. This technique, using a diagram with concentric circles around the participant, is described by Philips et al. (2000), referring to Kahn and Antonucci (1980) who first used this technique. We made some adaptations (e.g., in the measure we used we did not include family and we did not determine a maximum of names). Thus, three concentric circles were placed around the name of the participant who then mapped his or her relations with friends, neighbours, colleagues, other acquaintances and professionals by pointing within which circle a particular network member should be placed. The more important the network member, the closer the name is written to the name of the participant. The ecogram we used is outlined in the MSNA manual (van Asselt-Goverts et al. 2012). Finally, a five-point “stairway” scale was used to measure the functional characteristics of the participant’s social network in terms of four dimensions of supportiveness: the higher the score, the higher the step on the stairway.

#### Satisfaction and Wishes with Regard to the Social Network

To assess the satisfaction and wishes of the study participants with regard to their social networks, a questionnaire was developed based on the so called “scaling questions” that have their roots in Solution Focused interviewing (de Jong and Berg 2008; Roeden et al. 2009). The questionnaire consisted of four questions on satisfaction: one question about the network in general (‘How satisfied are you with your social network?’) and one question about satisfaction with respect to each of the three groups in the network in particular (‘How satisfied are you with your network of family/acquaintances/professionals?’). Responses were provided along a five point scale, ranging from very dissatisfied (score 1) to very satisfied (score 5). The five response possibilities were visualized as the five steps of a stairway, as also used in the MSNA. Next, we pointed at the stairway and asked the participant ‘What would make the satisfaction with your network one step higher?’ The answers of the participants gave us insight into their wishes with regard to their social network.

### Procedure

The scientific and ethics committee from *Dichterbij*, one of the organizations participating in this research, approved the present study. All 105 participants agreed to participate and provided written consent. Interviews were conducted by students at the HAN University of Applied Sciences and social workers from MEE support agencies in the Netherlands. Both groups were trained on how to administer the questionnaires. At the start of the interview, the participant was informed about the aims of the study, that all responses would be handled anonymously and that it was possible to stop the interview at any point. To enhance the reliability of data collection, an interview protocol and accompanying instruction manual was used (van Asselt-Goverts et al. 2012). The interviewers were trained in the use of the protocol and how to conduct an interview. The interviews were voice recorded, and the responses of the participants were also noted during the interviews.

### Data Analysis

The data were processed and analysed using SPSS (Version 20). To map the social networks of the participants, both the total network and the different groups within the network were analyzed: family (i.e., partner, children, parents, brothers/sisters and other family members); acquaintances (i.e., friends, colleagues, neighbours and other acquaintances) and professionals. Network members were included in the analyses if they were over the age of 12 years. With respect to wishes, the first expressed wish was coded and categorized further. Decisions concerning the coding and categorization of the wishes were discussed among researchers in the research group.

In the analyses several steps were undertaken. First, mean scores were calculated with regard to the structural characteristics (i.e., size of the social network, frequency of contact, initiation of contact and length of the relationships) and the functional characteristics (i.e., affection, connection, preference and practical/informational support). In a previous article these analyses are described in detail (van Asselt-Goverts et al. 2013). Second, the satisfaction of the participants with their current social networks, and their wishes with regard to these current social networks were determined. Percentages were calculated for this purpose. Third, in order to investigate whether the three groups (ID, ASD and REF) had different social networks, one way ANOVA’s (GLM) were performed for continuous outcome variables (i.e., for the structural and functional characteristics) and Chi Squared for categorical variables (i.e., for satisfaction and wishes). When applicable, Post hoc comparisons were conducted to determine which groups differed.

## Results

### Structural Characteristics of the Social Networks

#### Size

Table 2 gives information on the size of the social networks (i.e., the number of network members). On analysis of the number of network members shown on the ecogram (i.e., the visualisation of the social network excluding family), there were several significant differences between the three groups. Post hoc comparisons showed that participants with ID or ASD had significantly less network members on the ecogram than participants of the REF group (respectively *p* < .001; *p* = .012). Concerning the average number of network members on the MSNA (i.e., people from both genogram and ecogram who were considered important enough to put them on the MSNA according to the participant), Table 2 also shows differences on all variables except for acquaintances. Compared to the REF group, participants with ASD had a smaller number of network members (*p* = .046), informal network members (*p* = .022) and family members (*p* = .013) on the MSNA. Participants with ID had more professionals on their MSNA than participants with ASD (*p* < .001) and the REF group (*p* < .001). In consequence, the proportion of acquaintances in the network of people with ID was lower than in the network of the REF group, *p* = .020, while the proportion of professionals was higher than in the REF group and the ASD group, *p* < .001.

**Table 2:** Size of the social network (mean, SD) of the ID, ASD and REF groups compared

	ID		ASD		REF		*F*	*p*
	Mean	SD	Mean	SD	Mean	SD		
Ecogram^a^	9.42	6.1	11.27	7.1	17.86	11.7	9.184	.000
All members on MSNA^b^	14.21	6.5	11.27	5.7	15.00	6.6	3.182	.046
Informal network on MSNA^b^	11.21	6.3	10.30	5.2	14.33	6.7	4.340	.016
Family on MSNA^b^	6.00	3.4	5.20	2.5	7.55	3.9	4.574	.013
Acquaintances on MSNA^b^	5.21	4.2	5.10	3.9	6.79	3.8	2.158	.121
Professionals on MSNA^b^	3.00	1.5	0.97	1.3	0.67	1.1	32.750	.000

^a^An ecogram is a visualization of the social network excluding family; ^b^ Not all network members of the genogram and ecogram are listed in the MSNA, only the people the participant considered to be important enough to list them on the MSNA

#### Frequency of Contact

Table 3 presents detailed information on face-to-face contact, contact by telephone and contact by internet in times per year. Only face-to-face contact with acquaintances and internet contact with acquaintances and professionals differed significantly for the three groups. Post hoc comparisons showed participants with ID having more face-to-face contact with their acquaintances compared to both participants with ASD (*p* = .042) and to the REF group (*p* = .003). Moreover, participants with ID had less frequent internet contact with their professionals than the REF group (*p* = .025).

**Table 3:** Frequency of contact (times per year; mean, SD) of the ID, ASD and REF group compared

	ID		ASD		REF		*F*	*p*
	Mean	SD	Mean	SD	Mean	SD		
Face-to-face								
Family	81.61	58.5	90.80	57.2	95.73	52.4	0.588	.557
Acquaintances	116.96	92.4	63.28	70.1	51.06	38.7	8.737	.000
Professionals	65.95	50.1	56.86	83.8	51.17	69.2	0.299	.743
Telephone								
Family	102.48	172.5	93.63	94.7	116.77	139.6	0.253	.777
Acquaintances	97.41	117.7	74.28	172.1	55.46	56.8	0.999	.372
Professionals	13.11	17.8	69.13	138.6	9.27	12.6	3.152	.051
Internet								
Family	23.67	44.6	31.14	39.9	21.82	22.5	0.648	.525
Acquaintances	52.78	67.9	64.13	93.2	26.36	30.5	3.156	.047
Professionals	2.02	4.0	19.04	28.8	17.22	22.0	3.781	.030

#### Length of Relationships

With respect to the length of the relationships with acquaintances, analyses showed differences (*F* (2, 97) = 8.289, *p* < .001). Participants with ID knew their acquaintances on average 5.71 years (SD = 4.9), participants with ASD 8.55 years (SD = 3.8) and participants in the REF group 10.04 years (SD = 4.4). Post hoc comparisons showed that participants with ID knew their acquaintances for a shorter length of time than participants with ASD (*p* = .048) and participants of the REF group (*p* < .001). No significant differences were found in the length of the relationships with professionals. Participants with ID knew them on average 3.19 years (SD = 2.3); participants with ASD 2.22 years (SD = 2.4) and participants of the REF group 2.03 years (SD = 1.6).

#### Initiation of Contact

The initiation of contact can be considered as reciprocal (i.e., both the participant and the network member initiate the contact), but it is also possible that the participant or the network member is the main initiator or that neither the participant nor the network member explicitly takes the contact initiative, according to the participant. Analyses revealed very clear differences between the ID, ASD and REF groups in their perception of the initiation, for both family and acquaintances. Post hoc analyses revealed that participants with ID or ASD described their initiative less often as reciprocal than the REF group; this holds for the family network and the network of acquaintances (all *p* ≤ .005). Participants with ID saw themselves more often as the main initiator, compared to the REF group, for the family network (*p* = .003) and the network of acquaintances (*p* = .019); while participants with ASD saw their network member more often as the main initiator compared to the REF group (for family not significant; for acquaintances *p* = .040). Participants with ID described more often than the REF group that neither they themselves nor the network member explicitly took the initiative; for acquaintances this difference was significant, *p* = .031. No other significant differences were found.

### Functional Characteristics of the Social Networks

In this section we analyzed the differences in the functional characteristics of the social network of the three groups. Table 4 displays these functional characteristics, namely affection, connection, preference and practical/informational support. The three groups differed with respect to (a) affection for family and professionals, (b) connection to family, (c) preference for professionals and (d) practical and informational support from acquaintances. Post hoc comparisons showed the following results. Regarding affection, participants with ID assigned significantly lower scores to their family than the participants in the REF group, *p* = .017, and higher scores to their professionals, *p* = .003; this latter was also true for participants with ASD compared to the REF group, *p* = .005. Next, participants with ID appeared to feel less connected to their network members compared to both participants in the REF group (*p* = .001) and participants with ASD (*p* = .025) and in particular to their family in comparison with participants in the REF group (*p* = .035). Moreover, both participants with ID and ASD had a higher preference for the contact of their professionals, compared to the participants in the REF group, respectively *p* = .009 and *p* = .020. Finally, the participants in the ASD group perceived less practical/informational support from their acquaintances compared to the REF group, *p* = .039; the difference between participants with ID and ASD with regard to this was only marginally significant, *p* = .053.

**Table 4:** Functional network characteristics (mean, SD) of the ID, ASD and REF groups compared

	ID		ASD		REF		*F*	*p*
	Mean	SD	Mean	SD	Mean	SD		
Affection								
Family	3.93	0.7	4.16	0.7	4.33	0.4	3.827	.025
Acquaintances	3.80	0.6	4.02	0.8	3.90	0.6	0.754	.473
Professionals	4.00	0.8	4.11	0.9	3.08	1.0	7.328	.001
Connection								
Family	2.91	0.8	3.06	0.8	3.35	0.6	3.484	.034
Acquaintances	3.32	0.8	3.55	0.7	3.65	0.6	2.188	.118
Professionals	2.35	1.1	3.00	1.5	2.71	1.0	1.417	.251
Preference								
Family	4.09	0.6	4.04	0.7	4.27	0.5	1.530	.221
Acquaintances	4.00	0.6	3.99	0.7	4.04	0.6	0.070	.932
Professionals	3.90	0.8	3.94	0.7	3.09	1.1	5.672	.005
Practical/informational								
Family	3.63	0.8	3.73	0.9	3.87	0.7	0.807	.449
Acquaintances	3.75	1.0	3.22	0.9	3.73	0.7	3.960	.022
Professionals	4.30	0.8	4.33	0.8	3.84	0.9	2.074	.134

### Satisfaction and Wishes with Respect to the Social Networks

#### Satisfaction

In Table 5, the degrees of satisfaction of the participants with respect to their social networks in general, but also regarding the family, acquaintances and professionals in their social networks in particular, are presented. From the five-point scale, scores of 1 and 2 were summed as indicators of “dissatisfied” and the same was done for scores 4 and 5 as indicators of “satisfied”. As can be seen in Table 5 the satisfaction scores regarding the total network and the network of acquaintances were differently distributed between the three groups. Further analyses showed that for the total network all groups differed from each other: participants of the REF group were significantly more often satisfied; participants with ID or ASD more often neutral and this latter group was also more often dissatisfied. Moreover, with respect to the satisfaction with the network of acquaintances, participants with ASD reported more often to be neutral or dissatisfied and less often to be satisfied compared to participants of the REF group.

**Table 5:** Satisfaction with the social network (%) of the ID, ASD and REF groups compared

	ID	ASD	REF	*χ* ^*2*^	*p*
Network total				30.358	.000
Dissatisfied	3.8	30.0	0.0		
Neutral	23.1	26.7	2.4		
Satisfied	73.1	43.3	97.6		
Family				5.457	.222
Dissatisfied	7.1	10.0	2.4		
Neutral	25.0	33.3	16.7		
Satisfied	67.9	56.7	81.0		
Acquaintances				9.456	.043
Dissatisfied	7.4	23.3	2.6		
Neutral	14.8	20.0	10.5		
Satisfied	77.8	56.7	86.8		
Professionals				6.309	.141
Dissatisfied	11.1	8.0	0.0		
Neutral	7.4	24.0	26.9		
Satisfied	81.5	68.0	73.1		

#### Wishes

Table 6 presents the wishes with respect to the total network and with respect to the networks of family, acquaintances and professionals separately. The wishes were expressed in response to the open-ended question ‘What would make your network one step higher?’, which was asked with regard to the total network, family, acquaintances, and professionals separately. A large number of the participants did not answer this question or reported having no specific wishes and were excluded from these analyses; for the ID group *n* = 17; for the ASD group *n* = 7 and for the REF group *n* = 9. The reasons for not replying were stated as they were already satisfied, could not come up with something during the interview or found the question too difficult to answer. As Peter,^1^ a 33 years old men with ASD said:

**Table 6:** Wishes with respect to the social network (%) of the ID, ASD and REF groups compared

	ID	ASD	REF	*χ* ^*2*^	*p*
Wishes total network	*n* = 16	*n* = 23	*n* = 33	10.878	.197
More frequent contact	18.8	8.7	33.3		
Better contact	37.5	21.7	21.2		
Expanded network	6.2	21.7	12.1		
Improved social skills	25.0	30.4	9.1		
Other wishes	12.5	17.4	24.2		
Wishes family	*n* = 20	*n* = 20	*n* = 29	15.550	.027
More frequent contact	50.0	15.0	37.9		
Better contact	15.0	60.0	17.2		
Expanded network	5.0	0.0	3.4		
Improved social skills	10.0	15.0	10.3		
Other wishes	20.0	10.0	31.0		
Wishes acquaintances	*n* = 18	*n* = 20	*n* = 22	15.687	.034
More frequent contact	22.2	10.0	36.4		
Better contact	44.4	15.0	13.6		
Expanded network	0.0	20.0	9.1		
Improved social skills	27.8	20.0	13.6		
Other wishes	5.6	35.0	27.3		
Wishes professionals	*n* = 15	*n* = 18	*n* = 5	8.624	.140
More frequent contact	26.7	0.0	20.0		
Better contact	40.0	44.4	40.0		
Expanded network	6.7	0.0	0.0		
Improved social skills	0.0	0.0	0.0		
Other wishes	26.7	55.6	40.0

Look, that’s just how it is. I don’t need that many friends … I don’t need to know everybody.

As can be seen in Table 6, the wishes with respect to family and acquaintances differed between the three groups. First, regarding the family, people with ID wished more frequent contact, while people with ASD desired better contact with them (e.g., better contact with brother, sister, of family in general, patch up quarrels in the family, more depth in relationships) instead of more frequent contact with them. In the words of Miriam, diagnosed with ASD, mother of three children, two also diagnosed with ASD:

More understanding and respect from my parents … I usually have a bad connection with my family. They do not understand me at all, but neither do they understand my children. They have too little knowledge of autism.

Second, also regarding their acquaintances, participants with ID had other wishes than participants with ASD or the REF group; they wished better contact (e.g., having similar interests, wanting more pleasant contact and/or being taken more seriously) with their acquaintances instead of other wishes (e.g., acquaintances dwelling more in the neighbourhood, feeling good, having more elbow-room for personal things). Jessica, a 23 years old women with ID said, concerning better contact with friends:

More real life contact would be nice. I do have contact via MSN, but I would like more normal [face-to-face] contact.

Regarding their network of acquaintances, people with ASD, more often than people with ID, said they wished to expand their network, for instance with a partner. Elizabeth, a 35 year old women with ASD told us how difficult it is to get to know more people:

I long for many more contacts, but there is so much fear if someone actually comes closer that you clam up and it usually goes wrong again … To say things wrong. Not to respond in time. Not to have an answer when it is expected from you.

## Discussion

This study provides a comprehensive comparison of the perceptions of people with mild ID, people with ASD and a reference group towards their social networks. We first discuss the hypothesis that the social networks of people with ASD or ID are smaller, and then describe both the similarities and the specific characteristics of the networks of both groups. We finish with a discussion of the implications and limitations of our findings.

### The Networks of People with ASD and Mild ID: Size, Similarities, Specific Characteristics

Size was investigated using an ecogram (i.e., outline of all acquaintances and professional network members) and the MSNA. People with ID and people with ASD had less network members on their ecograms compared to the REFgroup, showing that their networks are more restricted. This is in line with previous research showing that the networks of people with ID are generally small (e.g., Robertson et al. 2001; Lippold and Burns 2009; Verdonschot et al. 2009) and that adults with ASD have fewer friendships (e.g., Howlin et al. 2004; Orsmond et al. 2004). Looking at the MSNA, in which important network members, according to the person, are listed and scored on a number of characteristics, a more detailed picture emerges. People with ASD have fewer informal network members listed on their MSNA compared to the REF group, especially fewer family members. On the other hand, people with ID have more professionals listed on their MSNA compared to both the ASD as the REF group. Remarkably, the people with ID did not have significantly fewer informal network members on their MSNA, although they did have fewer members on their ecogram. This can be explained by the fact that the people with ID put almost all network members from the ecogram on their MSNA, whereas people with ASD, and especially people from the REF group, were more selective. This emphasizes the statement in the Introduction section that the measures used are of importance in calculating the size of a social network. Due to its comprehensiveness, the MSNA seems to measure the quality of the most important relationships more than the actual size of the network. In future research we recommend using both the MSNA and the ecogram. In this study the family was mapped in a genogram and not included in the ecogram. In future research it is also recommended to add important family members to the ecogram, in order to get a complete and accurate picture of the social network size.

In addition, other network characteristics, satisfaction and wishes with respect to the network were compared, showing both similarities and differences. Both people with ID as people with ASD felt greater affection and preference for their professional network members compared to the REF group. This can be explained by differences in the nature of this professional support. For people with ID and ASD this support is necessary for daily life, while the REF group often meant the manager or supervisor at work. In actual practice it is important that staff members are aware of their importance in the lives of people with ID or ASD. People with ID or ASD were less often satisfied with their network and more often neutral than the REF group.

Although people with ASD varied widely in their perceptions of the quantity and meaning of their social connections, there were some common factors. People with ASD were more often dissatisfied, especially with their network of acquaintances. People with ASD experienced less practical and informational support from their acquaintances. They wished to expand their network of acquaintances and to improve the quality of their contact with family, instead of having more frequent contact with them. People with ASD saw their acquaintances as main initiators of the contact. A possible explanation is that for many of them, the inability to initiate contact is at the heart of their autistic disorder (American Psychiatric Association 2013). Indeed, they often wished they had better social skills.

In contrast, people with ID knew their acquaintances for a shorter duration, but saw them more often, compared to both the ASD and the REF group and they wished to improve these contacts. They felt less affection from and connection with family members and wished to have more frequent contact with them. Moreover, people with ID had the feeling that they were the main initiators of their contacts with their network members. The combination of their wish to have more frequent contact and a small network with which they already had high frequency contact might be an explanation of their perception that these network members less often took initiative.

### Limitations of the Study

Some limitations restrict the interpretation of our findings. First, the inclusion criteria (e.g., young adults, living independently in the community) may limit generalisation of the findings to younger or older people or people with more severe ID or ASD symptoms or to people living in group homes or with their parents. For instance, research shows that high-functioning adults with ASD are living with their parents in more than 50 % of cases (Renty and Roeyers 2006), so it is possible that the participants in our sample had better social skills than other high-functioning adults with ASD. The variation of the sample sources between the groups in this study was another potential limitation. The finding that the ID group has more professionals in their networks is possibly due to the fact that the ID participants were recruited via care organizations, from which they still received mobile support, while the ASD participants were recruited from a support agency giving support or advice.

Next, data were collected using self-report measures. Although it is possible that people with ID or ASD see themselves as more or less socially involved than others would report (Kasari et al. 2011), the use of proxies also has disadvantages. According to Verdugo et al. (2005), proxies should only be used when absolutely necessary, due to significant communication limitations which was not the case in this study. We tried to increase the reliability of self-reports of people with ID or ASD by adapting the measures, by simplifying the questions and by using visualization. Although we tried to ensure that the questions were not too difficult, in the section on wishes several participants couldn’t give an answer or specific wishes. Although it is possible that they indeed did not have any wishes, we have to consider the possibility that for some participants these questions were too complicated or too abstract. Overall, future research with other groups of participants is recommended. Gathering additional data from proxies is also recommended, when future results involves people with more severe ID or ASD.

Moreover, we did not focus on stressful characteristics of the network members, such as conflicts or the presence of ID, ASD or behavioural problems in network members. As such, network members can have a harmful rather than a beneficial influence (Lunsky and Haverkamp 1999). It is important to focus more on these issues, because it provides insight into the vulnerability of the network.

In this type of research it is always a challenge to obtain data from a sample size large enough to have sufficient power. Our sample size of 105 spread over three groups (ID, ASD and the reference group) gave a power of .80 and an effect-size of .30. This is slightly higher than .25, which is classified as a medium effect by Cohen (1992). Because differences with a small effect will not have been picked up in this study, we recommend repeating the study with a larger sample size.

Finally, this study does not indicate whether social inclusion for people with ID or ASD living in the community is a realistic possibility. Can network interventions alter social networks? In what way does training about networks affect the lives and social networks of people with disabilities? Relevant questions, requiring future research, because there is a critical need for evidence-based interventions to address social inclusion (Friedman et al. 2013).

### Practical Implications of the Study

It has been shown that social support benefits both physical and mental health and is related to lower rates of morbidity and mortality in the general population (e.g., Cohen and Wills 1985; Holt-Lunstad et al. 2010; Umberson and Montez 2010). Although there is no evidence yet for this benefit in people with ID (Emerson and Hatton 2008; Hulbert-Williams et al. 2011), associations between social support and quality of life for adults with ASD (Khanna et al. 2014; Renty and Roeyers 2006), for parents of people with ASD (Benson 2012; Pozo et al. 2013) and for adults with ID (van Asselt-Goverts et al. 2014b; Miller and Chan 2008; Bramston et al. 2005; Lunsky and Benson 2001) have been shown. For people with ASD, comorbidity with psychiatric disorders, such as mood and anxiety disorders, is very common (Hofvander et al. 2009; Mazzone et al. 2012; Seltzer et al. 2004). Moreover, people with ASD report lower health related quality of life than the general population (Khanna et al. 2014) and people with ID experience health inequalities (Emerson and Hatton, 2008). In the onset, expression and severity of these mental health problems, the environmental context may play an important role and social support might contribute to a decrease of these problems (Mazzone et al. 2012). Increasing health through social network enhancement might save health care expenses. This underlines the importance of social network interventions for people with ASD and ID.

Although both people with ID and people with ASD experience difficulties in developing and maintaining social contacts, the present research shows that each group has its own issues with regard to social network characteristics, satisfaction and wishes. Support staff should adapt to these network characteristics and to the needs and wishes with respect to the social networks to facilitate their social inclusion and as a consequence enhance their quality of life. For instance, in actual practice it can be useful to explore the reasons for a client perceiving him/herself or the network member as the main initiator of contact and support him/her to a more reciprocal initiation of these contacts. To adapt to network characteristics it is also recommended to use, in day-to-day practice, both the MSNA and the ecogram, because both measures have merits and limitations. In addition, the measure of satisfaction and wishes used in this research would also be useful for support staff. To facilitate social inclusion, the training of professionals may be necessary, for instance along the lines of Person Centered Planning (PCP; O’Brien et al. 2010). Because research shows that people with ASD are less likely to have a PCP plan (Claes et al. 2010; Robertson et al. 2007), future research on PCP with people with ASD is recommended. In the Netherlands an equivalent of PCP is available for people with mild ID; in this training offered by a self-advocacy group, they learn to map their network, their dreams and goals, their gifts, strengths and talents and to plan a meeting with network members (Blommendaal and van de Lustgraaf 2006). Because, in actual practice, it is a challenge to strengthen and expand the social networks, such training for professionals or clients should be followed by coaching (van Asselt-Goverts et al. 2014a). Moreover, these social network interventions should be examined for effectiveness, which is still an almost unexplored area in the care for people with ID and ASD.

## Footnotes

1 For the sake of anonymity, pseudonyms are used.
